# Astrocytoma progression scoring system based on the WHO 2016 criteria

**DOI:** 10.1038/s41598-018-36471-4

**Published:** 2019-01-14

**Authors:** Zhen-Hang Li, Yan-Lei Guan, Qiang Liu, Yao Wang, Run Cui, Yun-Jie Wang

**Affiliations:** Department of Neurosurgery, The First Hospital of China Medical University, No. 155, Nanjing North Street, Heping District, Shenyang, Liaoning Province 110001 PR China

## Abstract

Diffuse astrocytoma (including glioblastoma) is morbid with a worse prognosis than other types of glioma. Therefore, we sought to build a progression-associated score to improve malignancy and prognostic predictions for astrocytoma. The astrocytoma progression (AP) score was constructed through bioinformatics analyses of the training cohort (TCGA RNA-seq) and included 18 genes representing distinct aspects of regulation during astrocytoma progression. This classifier could successfully discriminate patients with distinct prognoses in the training and validation (REMBRANDT, GSE16011 and TCGA-GBM Microarray) cohorts (*P* < 0.05 in all cohorts) and in different clinicopathological subgroups. Distinct patterns of somatic mutations and copy number variation were also observed. The bioinformatics analyses suggested that genes associated with a higher AP score were significantly involved in cancer progression-related biological processes, such as the cell cycle and immune/inflammatory responses, whereas genes associated with a lower AP score were associated with relatively normal nervous system biological processes. The analyses indicated that the AP score was a robust predictor of patient survival, and its ability to predict astrocytoma malignancy was well elucidated. Therefore, this bioinformatics-based scoring system suggested that astrocytoma progression could distinguish patients with different underlying biological processes and clinical outcomes, facilitate more precise tumour grading and possibly shed light on future classification strategies and therapeutics for astrocytoma patients.

## Introduction

Gliomas are brain tumours that originate from glial cells. Star-shaped glia cells are called astrocytes, and tumours derived from these cells are referred to as astrocytomas. The 2016 WHO classification of diffuse astrocytic tumours indicates three grades with different aggressiveness^1^. Compared to the other types of gliomas, diffuse astrocytomas present unique challenges for treatment due to their heterogeneity, aggressive biological behaviour and diffusive growth. Diffuse astrocytomas are comprised of two major subtypes according to the newest WHO criteria: IDH-mutant and IDH-wild type (diffuse astrocytoma, anaplastic astrocytoma and glioblastoma).

The modern diagnosis of astrocytoma relies on computer-assisted tomography (CT) and magnetic resonance imaging (MRI). These techniques are indispensable for determining the localization and size of the lesions. Radiological features that can differentiate an astrocytoma from other brain parenchymal lesions have also been described^2,3^, but in most cases the accuracy of the radiological features is not sufficient to make clinical management decisions. Cytological and histopathological analyses of bioptates collected during brain surgery or biopsy have long been the only choice for long-term, reliable establishment of glioma grading. These considerations indicate a clear need for accurate and robust biomarkers, which may help determine the astrocytoma prognosis and grading.

An astrocytoma originates from complex interactions between developmental and genetic factors, which leads to heterogeneity across patients. To date, the transcriptomic classification divides astrocytomas into different subtypes, including classical (CL), neural (NE), mesenchymal (ME), and proneural (PN)^4^. Great efforts have been made to identify molecular markers for prognostic prediction. Many recent studies have focused on gene expression profiles in glioma to construct signatures^5–10^, which have shown great promise for prognostic prediction in individual patients. However, the limitation of the gene selection method, which uses formula designation for application within different platforms, is also obvious. Thus, identifying a more powerful and practical scoring system for the heterogeneity and intrinsic characteristics of the tumour for prognostic prediction has great clinical significance.

In the current study, with an aim of developing a scoring system for astrocytoma progression and prognosis, we carried out a supervised approach associated with the astrocytoma grade and clinical outcome. Following this principle, we performed a combined analysis to identify two robust gene sets related to astrocytoma progression and established a scoring system.

## Results

### Selection of astrocytoma progression-related genes and construction of the AP score

**Figure 1:**
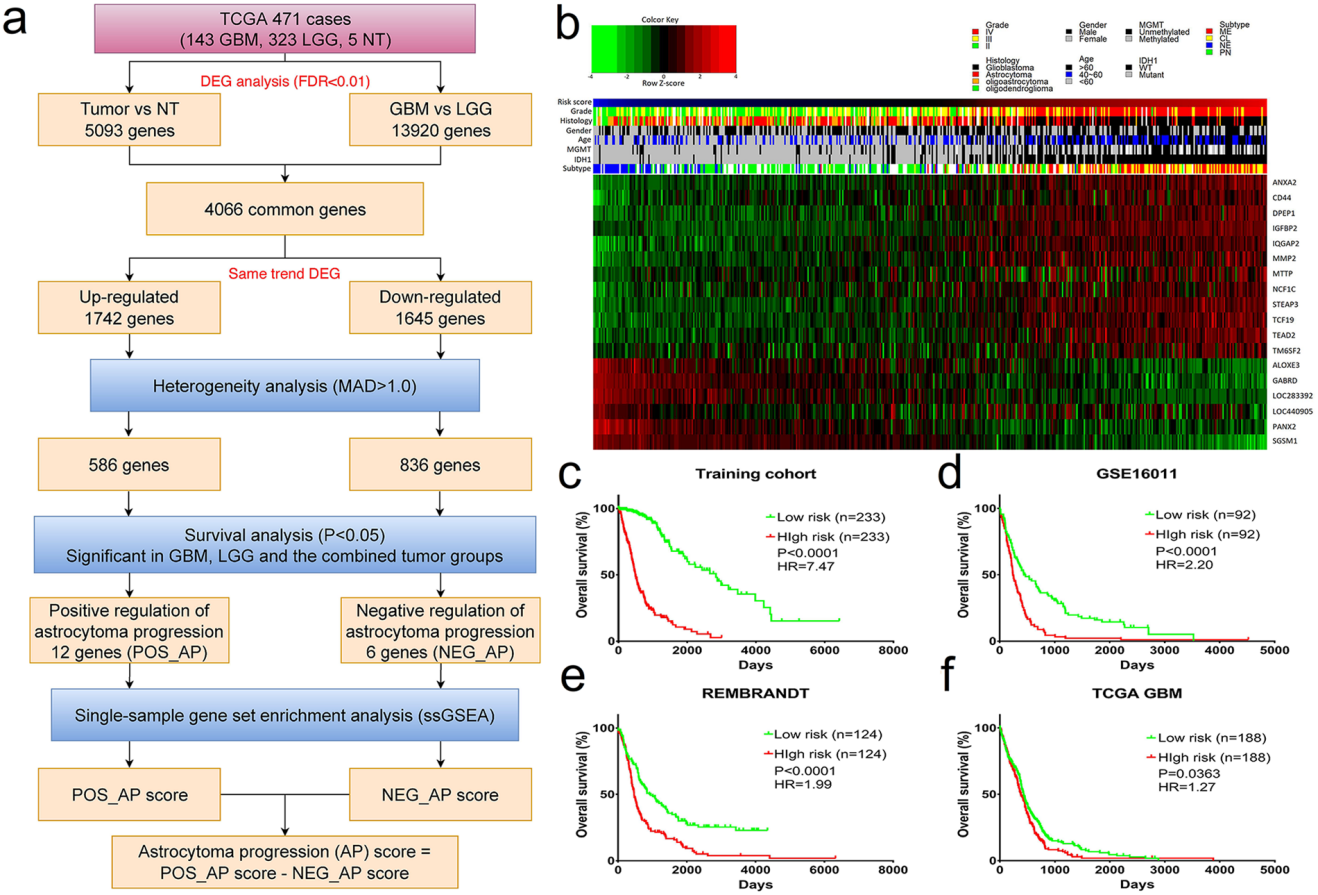
Development of the AP score and its prognostic value across cohorts. (**a**) An overview of the AP score algorithm. The AP score algorithm uses gene expression data to output the combined degree of positive and negative regulation of astrocytoma progression. (**b**) Heatmap depicting the Z-score expression values of the 18 genes in the training cohort. Columns represent each sample and are labelled with their clinical characteristics, and rows represent genes and are divided into two groups representing POS_AP and NEG_AP. (**c**) Kaplan-Meier survival analyses based on the median cutoff AP score in the training dataset. (**d**) Kaplan-Meier survival analyses based on the median cutoff AP score in the GSE16011 dataset. (**e**) Kaplan-Meier survival analyses based on the median cutoff AP score in the REMBRANDT dataset. (**f**) Kaplan-Meier survival analyses based on the median cutoff AP score in TCGA GBM dataset. Mutant: IDH1 mutant, WT: IDH1 wild type; NE: Neural, PN: Pro-neural, CL: Classical, ME: Mesenchymal.

We obtained gene expression profiles for 466 astrocytomas (323 LGG and 143 GBM) and 5 NTs from TCGA. We obtained 30 genes for analysis that met the following filtering criteria (Fig. 1a): (i) genes were differentially expressed between tumour and normal tissue, (ii) genes were differentially expressed between GBM and LGG tissue, (iii) genes demonstrated highly variable expression profiles (median absolute deviation, MAD > 1.0), (iv) genes with the same DEG trend, and (v) genes related to patient survival (Kaplan-Meier log-rank test p < 0.05). Thus, the resulting 18 genes (POS_AP: ANXA2, CD44, DPEP1, IGBP2, IQGAP2, MMP2, MTTP, NCF1C, STEAP3, TCF19, TEAD, and TM6SF2; NEG_AP: ALOXE3, GABRD, LOC293392, LOC440905, PANX2, and SGSM1) not only represented genes that were significantly related to astrocytoma initiation and progression but also genes that exhibited heterogeneity among tumour samples and in terms of the prognosis (Fig. 1a,b).

To investigate the roles of the 18 genes in the regulation of astrocytoma progression, we nominated upregulated genes as positive regulators of astrocytoma progression (POS_AP) and downregulated genes as negative regulators of astrocytoma progression (NEG_AP).

Based on the two distinct gene sets, we employed the ssGSEA method to construct a scoring system for analysis, and the astrocytoma progression (AP) score was set as follows: AP score = POS_AP score − NEG_AP score.

### The prognostic value of the AP score across different cohorts

**Figure 2:**
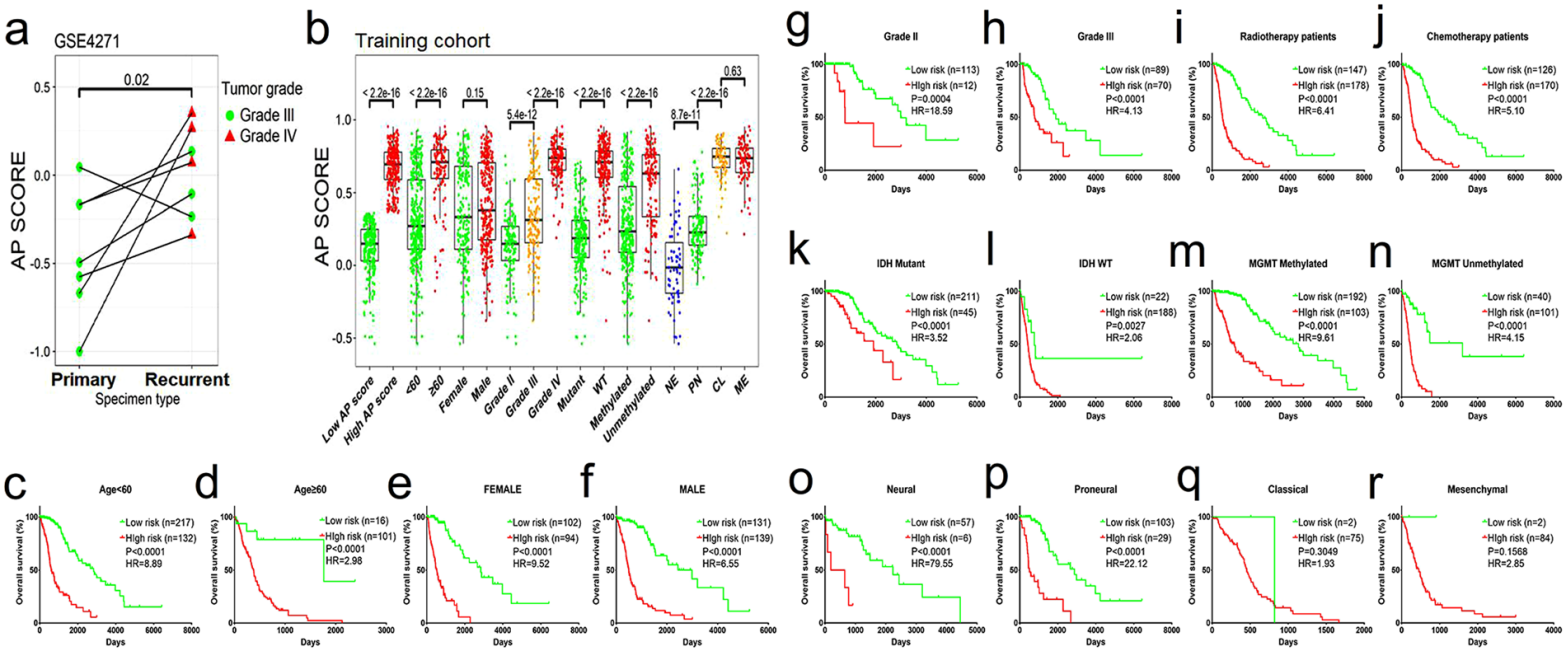
The AP score in the longitudinal dataset and the prognostic value of the AP score across different subgroups of the training cohort. (**a**) The AP score in subgroups of primary-recurrent paired astrocytomas in the GSE4271 cohort. (**b**) Distribution of the AP score among astrocytoma subgroups in the training cohort. ANOVA p < 0.001 in the grade and subtype subgroups. (**c**–**r**) Kaplan-Meier survival analyses based on the dichotomized AP score in the supergroups defined by clinicopathological factors of the training cohort.

To explore whether the AP score was related to astrocytoma patient survival, first we analysed the training dataset (TCGA RNA-seq cohort), which contained 466 cases. The samples were dichotomized into either high (n = 233) or low (n = 233) subgroups classified by the median AP score. Our analyses demonstrated that a low AP score was a potent independent marker for predicting better overall survival in the training dataset (median survival: 2835 days for the low AP score group vs 492 days for the high AP score group, *P* < 0.0001, HR = 7.47; Fig. 1c). We obtained similar results when analysing the GSE16011 (*P* < 0.0001, HR = 2.20; Fig. 1d) and REMBRANDT cohorts (*P* < 0.0001, HR = 1.98; Fig. 1e). These results demonstrated that the AP score was a significant reliable marker for predicting the prognosis of astrocytoma patients. To the best of our knowledge, a longitudinal dataset comprised of primary-recurrent astrocytoma is most relevant for disease progression. To validate the AP score concept, we employed the GSE4271 dataset and filtered it according to the criterion that a primary astrocytoma should be a lower-grade astrocytoma. Seven paired astrocytomas fit the criterion. Then, the AP score was calculated for the 14 astrocytomas (Fig. 2a). The AP score of the recurrent group was significantly higher than that of the primary group (t test, *P* = 0.02). A total of 6/7 of the tumours had an elevated AP score when recurrent (grades III to IV: 4/7; grades III to III: 2/7). The left one that showed a decreased AP score did not progress to grade IV GBM when recurrent.

### Distribution and prognostic value of the AP score among the astrocytoma subgroups

A related question is whether the associations between the AP score and the clinicopathological parameters and molecular features reflect the differences between glioblastoma and low-grade tumours. These conditions are so unique that any reasonable strategy will find genes that can distinguish them. To validate this question, the patients in the training cohort were stratified based on several clinicopathological factors, including age, gender, grade, received therapy, IDH mutation status, MGMT methylation status and transcriptional subtypes. We found that patients with an older age, higher grade, IDH wild type and unmethylated MGMT status demonstrated higher AP scores, whereas gender conferred little impact on the AP score distribution. With respect to transcriptional subtypes, mesenchymal and classical subtypes had relatively high AP scores, neural subtypes had the lowest AP scores, and the proneural subtype had intermediate scores (Fig. 2b). Based on the median cut-off value of the AP score in the training cohort, the patients were dichotomized into either high or low supergroups within each subgroup. A high AP score accounted for a significantly high proportion of the patients with an older age, grade IV, IDH1 wild type, unmethylated MGMT status, and classical and mesenchymal subtypes, whereas a low AP score accounted for a significantly high proportion of the patients with a younger age, grades II and III, IDH1 mutant, methylated MGMT status, and neural and proneural subtypes. No significant difference was observed between the female and male patients (Supplemental Table S1). We applied the dichotomized AP score for the whole cohort to all subgroups to query the prognostic value. A nearly universal result was achieved, demonstrating that a high AP score was highly correlated with a poor prognosis and vice versa (Fig. 2c–r). With respect to the tumour grade, obtaining a significant prognostic value was difficult for grade IV cases (low AP score n = 4, high AP score n = 139). Here, a TCGA microarray dataset comprising GBM patients (all cases with wild type IDH) was used for validation (*P* = 0.0363, HR = 1.27; Fig. 1f). Univariate and multivariate Cox regression analyses also indicated that the AP score was an independent prognostic factor after adjusting for other clinical covariates (Table 1).

**Table 1:** Univariate and multivariate Cox regression analyses in the training cohort.

Variables	Univariate COX		Multivariate COX	
	HR	P	HR	P
AP score (High vs Low)	7.87	**<0.0001**	2.68	**<0.0001**
Disease (GBM vs LGG)	7.40	**<0.0001**	1.98	**0.0004**
Age (≥60 vs <60)	4.38	**<0.0001**	1.50	**0.0310**
Gender (Male vs Female)	1.19	0.2295	—	—
MGMT (Unmethylated vs Methylated)	2.93	**<0.0001**	1.32	0.0996
IDH1 (Wild type vs Mutant)	10.39	**<0.0001**	3.54	**<0.0001**
Chemotherapy (Yes vs No)	0.68	**0.0206**	0.98	0.9239
Radiotherapy (Yes vs No)	0.92	0.5906	—	—

### The AP score reliably predicted patient outcomes

Receiver operating characteristic (ROC) analysis showed that the areas under the receiver operating characteristic curve (AUCs) for the tumour grade (GBM vs lower grade astrocytoma), MGMT promoter methylation status, IDH mutation status, AP score and combined factor of the IDH status and AP score were 0.7543, 0.6509, 0.8220, 0.8367 and 0.8513, respectively, in the training cohort (Supplemental Fig. S1). The results suggested that the AP score performed as well as the IDH mutation status in predicting patient outcomes and achieved an even higher prediction value when combined with the IDH mutation status.

### The AP score was associated with distinct patterns of somatic mutations and copy number variation

To further investigate the impact of the AP score in the DNA level scenario, TCGA cases with available somatic mutation (460/466) and copy number variation (CNV) information (464/466) were analysed. The cases were divided into two subgroups based on the increase in the AP score (the lower and higher AP score groups).

**Figure 3:**
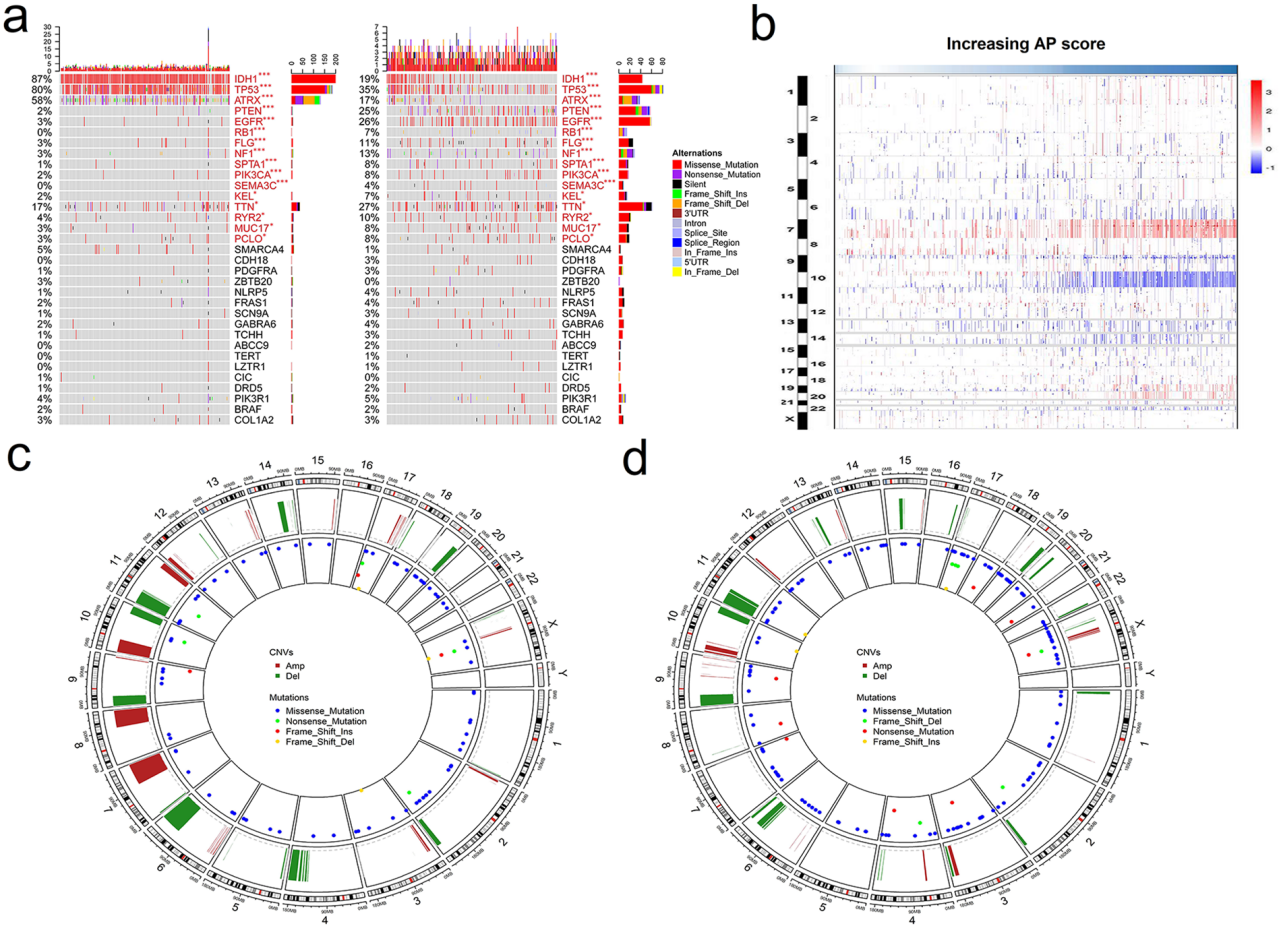
Different mutation and copy number variation patterns of the AP score. (**a**) Summary of well-known individual regulators of glioma from 460 samples from the training cohort. Columns are sorted by samples with increasing AP scores. Top histogram, the sum of mutations in each sample category is indicated by the legend; Right histogram, the sum of mutations in each gene is indicated by the legend. (**b**) The overall copy number variation (CNV) profile in order of increasing AP score. (**c**,**d**) A distinct CNV and recurrent mutation profile is observed between astrocytomas with low and high AP scores. *P < 0.05; **P < 0.01; ***P < 0.001.

The mutation status of well-known individual regulators of glioma was analysed (Fig. 3a). Frequent mutations in IDH1, ATRX and TP53 were significantly enriched in cases with lower AP scores, whereas PTEN, EGFR, RB1, FLG, NF1, SPTA1, PIK3CA, SEMA3C, KEL, TTN, RYR2, MUC17 and PCLO were significantly enriched in cases with higher AP scores.

Subsequently, CNV data were investigated and showed distinct chromosomal alteration patterns between astrocytomas with low and high AP scores. Chr 7 amplification paired with Chr 10 loss was enriched in the higher AP score cases (Fig. 3b). In the gene level CNV landscape, the frequently deleted genomic regions were 9p21.3 encompassing CDKN2A/CDKN2B (mean deletion, CDKN2A −0.191 vs −0.745, *P* < 0.001; CDKN2B −0.191 vs −0.729, *P* < 0.001) and 10q23.3 encompassing PTEN (−0.058 vs −0.635, *P* < 0.001). Conversely, 7p11.2 encompassing EGFR (mean amplification, 0.195 vs 1.836, *P* < 0.001), PDGFRA (4q12; 0.045 vs 0.420, *P* < 0.001) and CDK4 (12q14.1; −0.029 vs 0.663, *P* < 0.001) were frequently amplified with higher AP scores (Fig. 3b–d and Supplemental Fig. S2).

### High AP score astrocytomas exhibited a malignant phenotype

**Figure 4:**
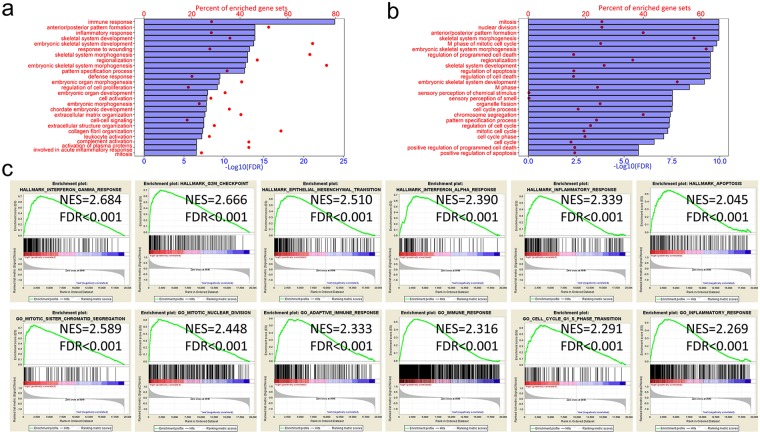
Biological function of the AP score. (**a**) The top 25 GO terms for upregulated DEGs [Log10(FC) > 1.5] enriched based on the AP score. (**b**) The top 25 GO terms of highly correlated (r > 0.6) genes enriched based on the AP score. (**c**) GSEA results based on the increasing AP score.

Regarding the prognostic value of the different mutation and CNV patterns based on the AP score, GO analysis was performed to explore the functional aspects. We performed the DEG analysis based on high and low AP scores and gained 1585 genes that were upregulated in the high AP score group at a FDR of 0.01 and the lowest log fold change (logFC) of 1.5 for the GO analysis. The results suggested that a high AP score was associated with immune/inflammatory responses and cell cycle-related processes (Fig. 4a). To validate the results, Pearson’s correlation score (r) was calculated for each gene in the training cohort. The GO results based on 1215 genes that were positively correlated (r > 0.6) with the AP scores suggested that the genes were highly enriched in cell cycle-related processes (Fig. 4b). The GO results for either positively correlated genes or differentially expressed genes were in great concordance with the cell cycle-related processes. Meanwhile, immune/inflammatory response genes were found in the DEG panel. The GO results based on the 822 downregulated genes in the high AP score group with an FDR of 0.01 and the lowest log fold change of −1.5 and the 1139 negatively correlated (r < −0.6) with the AP score suggested that these genes were highly enriched in relatively normal nervous system functions (Supplemental Fig. S3). The GSEA analysis further validated that the AP score was associated with processes or pathways that were closely related to immune/inflammatory responses and the cell cycle (Fig. 3c and Supplemental Fig. S3). For gene sets that were enriched in the GSEA analysis, ssGSEA was performed in the training dataset. Pearson’s correlation scores were calculated based on the ssGSEA value of the gene sets queried in the GSEA and the AP score. We observed high correlations between the gene sets and AP score. The gene sets could be inferred to be of high importance in regulating astrocytoma progression. To validate this hypothesis, the samples were dichotomized into either high or low subgroups classified by the median ssGSEA score calculated from each gene set. The log-rank test results based on the classification demonstrated that all gene sets had significant prognostic value (Supplemental Table S2).

### Association between the AP score and immune cells

The cell cycle and apoptosis are among the most well understood processes regulating glioma progression and the prognosis, whereas the immune/inflammatory response has only recently been identified as a key player in regulating glioma tumourigenesis and the prognosis. Thus, we performed ssGSEA with gene sets comprised of mRNA transcripts specific for most innate and adaptive immune cell subpopulations. The gene sets were representative of immune cells that constituted the “immunome”. The genes in each gene set were most representative of purified immune cells^11^. The correlation and prognostic value of each immune cell were also calculated (Table 2). Macrophages, activated dendritic cells (aDCs), NK CD56dim cells, NK cells, T helper cells (Th2), eosinophils, immature dendritic cells (iDCs), neutrophils, T cells, dendritic cells (DCs), T helper cells and T helper 1 (Th1) cells, most of which represented the innate immune system, were positively correlated with the AP score and gained significant prognostic value when dichotomized by the median ssGSEA score. T central memory (Tcm) cells, T gamma delta (Tgd) cells, B cells and T effector memory (Tem) cells, most of which represented the adaptive immune system, were negatively correlated with the AP score and gained significant prognostic value. Therefore, most immune cells with significant prognostic value were associated with the AP score.

**Table 2:** Correlation and prognostic value of each immune cell in the training cohort.

Immune cell	Log-rank test	Correlation with AP score (Pearson correlation)	
	P	r value	P
Macrophages	<**0.001**	0.712	<**0.001**
aDC	<**0.001**	0.640	<**0.001**
NK_CD56dim	<**0.001**	0.543	<**0.001**
NK	<**0.001**	0.449	<**0.001**
Th2	<**0.001**	0.408	<**0.001**
Eosinophils	<**0.001**	0.385	<**0.001**
iDC	**0.012**	0.356	<**0.001**
Neutrophils	<**0.001**	0.337	<**0.001**
T_cell	<**0.001**	0.321	<**0.001**
DC	**0.029**	0.271	<**0.001**
Th17	0.385	0.182	<**0.001**
TReg	0.161	0.164	<**0.001**
T_helper	**0.042**	0.163	<**0.001**
Th1	**0.023**	0.104	**0.024**
Cytotoxic	0.551	0.048	0.301
CD8	**0.001**	−0.003	0.950
pDC	<**0.001**	−0.083	0.072
Mast	0.841	−0.107	**0.021**
NK_CD56bright	0.614	−0.219	<**0.001**
Tem	<**0.001**	−0.303	<**0.001**
B	**0.005**	−0.309	<**0.001**
Tgd	<**0.001**	−0.316	<**0.001**
Tcm	<**0.001**	−0.418	<**0.001**

## Discussion

As a common fatal central nervous system tumour, astrocytoma is diagnosed by genotype and histopathological criteria, although the genotype always trumps the histological phenotype^1^. Diffuse astrocytomas show relentless and malignant progression characterized by extensive invasion throughout the brain. Most patients with a low-grade astrocytoma progress to a highly malignant astrocytoma or glioblastoma. However, there is accumulating evidence that tumours with similar histology have distinct molecular signatures that significantly impact the treatment response and survival. Additionally, malignant advances occur within the same tumour grade, although this phenomenon has been little studied. Robust prognostic evaluation of most tumour-related factors is limited to the grade and genetic variation. Recently, the molecular classification of astrocytomas has developed rapidly. Several classification systems based on mRNA expression^4,12^ were established to classify tumours with equal grades into several subtypes. As tumour malignancy progresses, the tumour subtypes can transit into each other, similar to the mesenchymal and proneural subtypes in GBM^13^. Understanding the mechanisms of grade progression and subtype transition and blocking the major oncogenic pathways are the keys to astrocytoma therapy^14^.

To discover key genes regulating the oncogenesis and malignant progression of astrocytomas, we performed bioinformatics analyses of RNA expression data and revealed 18 key regulator genes corresponding to 2 subgroups, including 12 genes that were upregulated as malignancy progressed and 6 genes that were downregulated. Although the genes were mainly filtered based on GBM vs LGG and tumour vs normal tissue given other restrictions, such as same DEG trend and the prognostic analysis, we assumed that the combined signature of the two scores mostly reflected astrocytoma progression. Further functional analysis also suggested that cases with higher AP scores were associated with processes conferring tumour malignancy, whereas lower AP scores reflected relatively normal CNS function, which met the concept of astrocytoma progression.

Increasing evidence suggests that genetic changes (mutations, deletions, amplifications and overexpression) are involved in the development and progression of gliomas^15^. Established astrocytoma biomarkers, such as TP53 and IDH1 mutations and the recently discovered ATRX mutations, are thought to be early events in these tumours^16^. In our analysis, TP53, IDH1 and ATRX were more likely to be mutated in the lower AP score group than in the higher AP score group, suggesting their roles in astrocytoma oncogenesis. However, more mutation in PTEN and EGFR were detected in the high AP score group. Mutations in the tumour suppressor gene PTEN are frequent events and are associated with therapeutic resistance, because PTEN is a key player in regulating glioblastoma oncogenesis^17,18^. Alterations in signature oncogenes of GBM, such as EGFR, always confer a worse prognosis^18,19^. Different CNV patterns were also observed, such as EGFR, PDGFRA, and CDK4 amplification and CDKN2A/CDKN2B and PTEN deletion. These results suggested that the AP score could exert its progression estimation value at the DNA level.

Regarding functional aspects, a lower AP score demonstrated relatively normal CNS function, whereas a higher AP score was associated with processes conferring tumour malignancy. Among the well-studied processes regulating glioma progression and the prognosis, the cell cycle plays a central role in development and carcinogenesis^20,21^. Accumulated evidence indicates that abnormal cell cycle progression may confer tumour advancement and radio-resistance^22^ of glioma cells. Numerous studies have elucidated the important role played by the tumour microenvironment in cancer progression. In particular, the formation of hypoxic regions within the enlarged mass of solid tumours and the consequent induction of angiogenic switches are key steps in glioma development and progression^23^. The CNS has been clearly shown to coordinate a robust immune response with the innate and adaptive immune systems rather than inducing immune privilege^24^. However, the general role of the local immune response in astrocytoma progression and the prognosis remains unclear. Several studies have focused on local immune phenotypes of glioma and have indicated that either an immune signature or immune cell enrichment may play a role in predicting the patient prognosis and tumour malignancy^25,26^, similar to the discoveries in our study. The relatively normal CNS processes associated with a low AP score also suggest that astrocytoma formation and progression occur at the cost of sacrificing regular CNS function while gaining malignant phenotypes.

In conclusion, our findings highlighted the important role of the AP score and its related processes in the biology and clinical management of astrocytoma. The astrocytoma progression score had a considerable impact on the clinical, genomic and biological status. Evaluating the progression score of astrocytoma may help elucidate the complex role of tumour malignant processes, facilitate further astrocytoma malignancy grading and provide new insights into clinical management and drug design.

## Methods

### Datasets

Whole genome mRNA expression RNA-seq, somatic mutation, and copy number variation data and corresponding clinical information, including the tumour subtype, IDH mutation status, 1p-19q co-deletion status, and MGMT promoter methylation status, were downloaded from TCGA dataset^16,18^ (http://cancergenome.nih.gov/) as the training cohort. According to the WHO 2016 criteria, adult diffuse glioma centres around isocitrate dehydrogenase (IDH) and 1p/19q diagnostics^1,27–29^. Thus, we designed the inclusion criteria based on the newest WHO classification, with the training cohort comprised of pure 1p/19q non-codeletion cases with a mutant or wild type (WT) IDH status. The following three datasets were obtained for validation: Repository for Molecular Brain Neoplasis Data (REMBRANDT, http://caintegrator.nci.nih.gov/rembrandt), GSE16011 (http://www.ncbi.nlm.nih.gov/geo/query/acc.cgi?acc = GSE16011)^30^, and GSE4271 (https://www.ncbi.nlm.nih.gov/geo/query/acc.cgi?acc = gse4271)^31^. Additionally, we included mRNA expression microarray data from TCGA glioblastoma multiforme (GBM) project^18^. Normalized expression data were downloaded from each data source. Due to the lack of precise identification of the 1p/19q status in the validation cohorts, we only included those who were diagnosed as astrocytoma or glioblastoma. The training dataset comprised 466 astrocytoma and glioblastoma samples and 5 normal tissue (NT) samples. The REMBRANDT and GSE16011 datasets comprised 248 and 184 astrocytoma and glioblastoma patients, respectively, whereas the TCGA microarray dataset comprised 376 glioblastoma patients. The patients’ characteristics are summarized in Supplemental Table S1.

### Statistical analysis

Overall survival (OS) was defined as the interval from the date of diagnosis to death or the last follow-up. The prognostic differences between patients with high or low expression of a certain gene or score (higher or lower than the median value) were calculated by the Kaplan–Meier method with the two-sided log-rank test using the R “survival” package. Univariate and multivariate COX regression analyses were also performed with the R “survival” package. The hazard ratio (HR) was calculated with the Mantek-Haenszel method in the GraphPad Prism 7.00 software or by the COX method in the R “survival” package using the Kaplan-Meier log-rank test or COX analysis, respectively. A two-tailed Student’s t-test was performed to compare two groups of numerical values. Analysis of variance (ANOVA) was used to analyse differences among group means. The Chi-square test and Fisher’s exact test were used to compare frequencies between groups. The R “pROC” package was used in the ROC analysis and for comparisons between factors. The generalized linear model was fitted when combining two factors. The prediction performance was evaluated with area under the receiver operating characteristic curve (AUC) estimation. For the ROC analysis, patients who were not censored at the last follow-up and those whose durations were less than the mean OS were excluded. Pearson’s correlation analysis was used to evaluate the association between two variables. The statistical analysis was performed with the R software version 3.51 for Windows. Statistical significance was set at the level of p < 0.05.

### Bioinformatics analysis

Level 3 RNA-seq data from TCGA-LGG and TCGA-GBM cases that met the inclusion criteria were downloaded and normalized within the R package “TCGAbiolinks”^32^. The “edgeR” package was used to identify differentially expressed genes (DEGs) based on the threshold of a false discovery rate (FDR) less than 0.01. The “TCGAbiolinks” package of R was applied to investigate relevant biological implications. The biological phenotype was further verified by gene set enrichment analysis (GSEA)^33^. Single sample GSEA (ssGSEA) of the enriched and immune cell gene sets was performed using the R gene set variation analysis (GSVA) package^34^. The gene list of immune cells was summarized by Gabriela Bindea *et al*.^11^.

## Electronic supplementary material

Supplementary Info

## Data Availability

The expression, CNV, somatic mutation and clinical data from 471 cases (training cohort) and 525 GBM cases belonging to TCGA dataset were retrieved from the website http://cancergenome.nih.gov/. The REMBRANDT dataset was retrieved from the website http://caintegrator.nci.nih.gov/rembrandt. The GSE16011 dataset was retrieved from the Gene Expression Omnibus website (http://www.ncbi.nlm.nih.gov/geo/query/acc.cgi?acc=GSE16011). The GSE4271 dataset was retrieved from the Gene Expression Omnibus website (https://www.ncbi.nlm.nih.gov/geo/query/acc.cgi?acc=gse4271).
