# Supplementary material for: Astrocytoma progression scoring system based on the WHO 2016 criteria
Source: Sci Rep. 2019 Jan 14;9:96. doi: 10.1038/s41598-018-36471-4 (PMC6331604; doi:10.1038/s41598-018-36471-4)
Supplement: Supplementary file 1 — Supplementary Info [file 41598_2018_36471_MOESM1_ESM.doc]

**Astrocytoma progression scoring system based on the WHO 2016 criteria**

Zhen-Hang Li, M.D., Ph.D. ^1^, Yan-Lei Guan, M.D., Ph.D.^1^, Qiang-Liu, M.D., Ph.D. ^1^, Yao Wang, M.M.^1^, Run Cui, M.D., Ph.D.^1^, Yun-Jie Wang, M.D., Ph.D.^1,*^

^1^ Department of Neurosurgery, The First Hospital of China Medical University, No.155, Nanjing North Street, Heping District, Shenyang, Liaoning Province 110001, PR China

^*^ **Correspondence**: Department of Neurosurgery, The First Hospital of China Medical University, No.155, Nanjing North Street, Heping District, Shenyang, Liaoning Province, PR China

Phone: +86 15942059310

E-mail address:

[Lizhenhang0510@outlook.com](mailto:Lizhenhang0510@outlook.com) (Z. –H. Li, **first author**)

[drguanyanlei@outlook.com](mailto:drguanyanlei@outlook.com) (Y. –L. Guan)

[liuqiang1221@hotmail.com](mailto:liuqiang1221@hotmail.com) (Q. Liu)

[mswangyao@outlook.com](mailto:mswangyao@outlook.com) (Y. Wang)

[cuirunrunrun@hotmail.com](mailto:cuirunrunrun@hotmail.com) (R. Cui)

[Dryunjiewang@hotmail.com](mailto:Dryunjiewang@hotmail.com) (Y. –J. Wang, **corresponding author**)


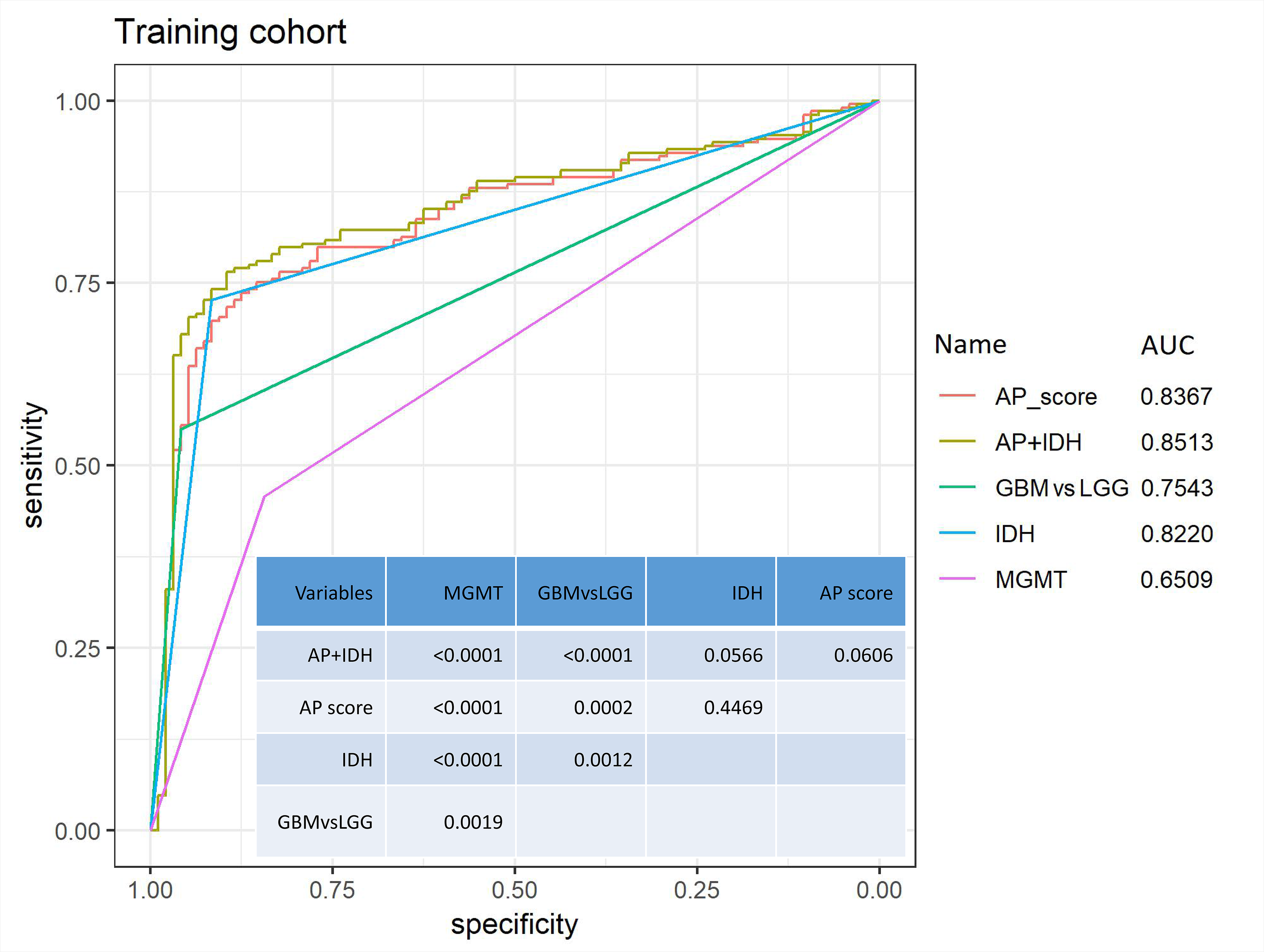


**Figure S1.** ROC analyses of AP score of the training cohort.


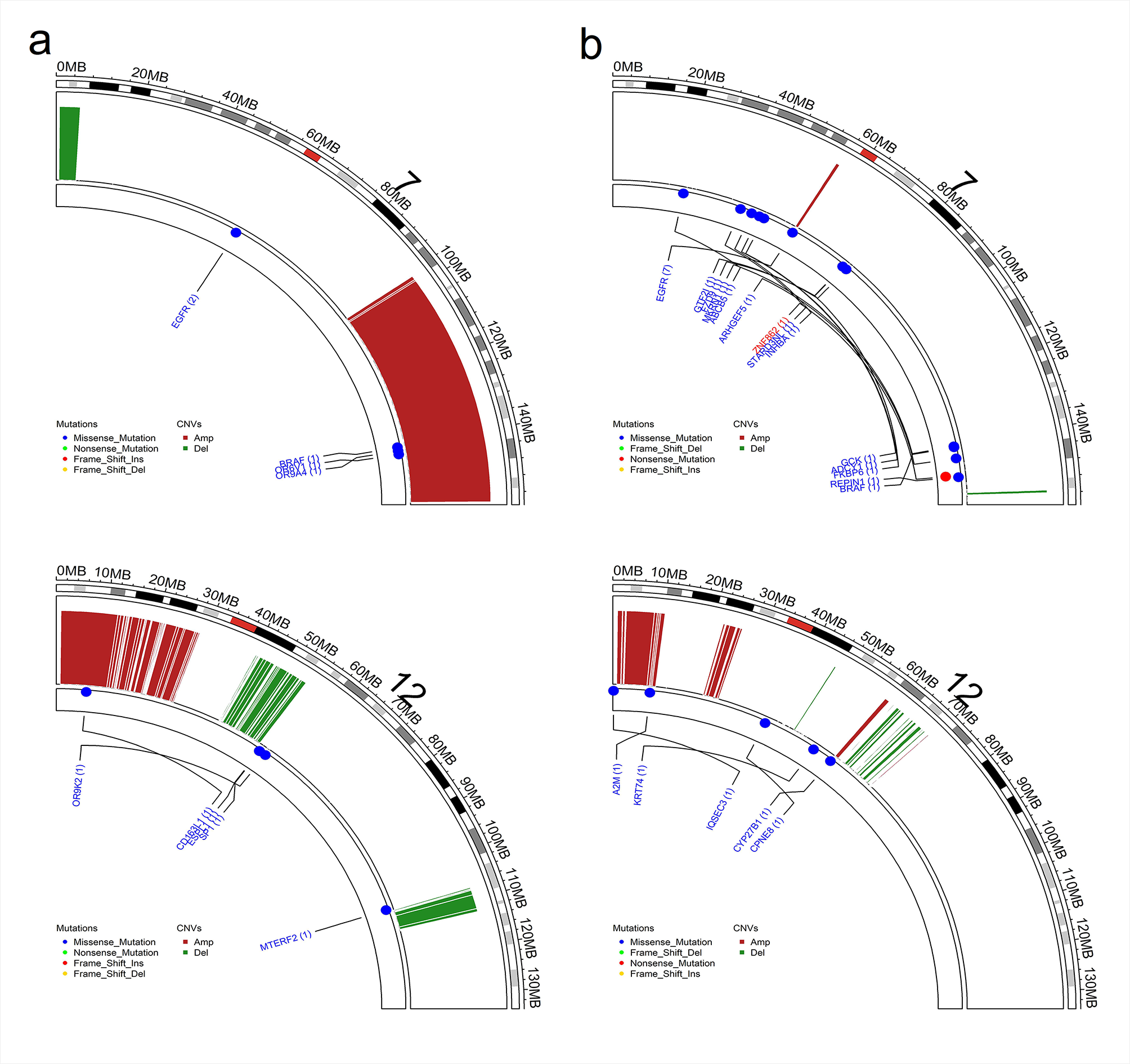


**Figure S2.** Detailed recurrent mutation and copy number variation pattern in lower AP score group **(a)** and higher AP score group **(b)**.

**
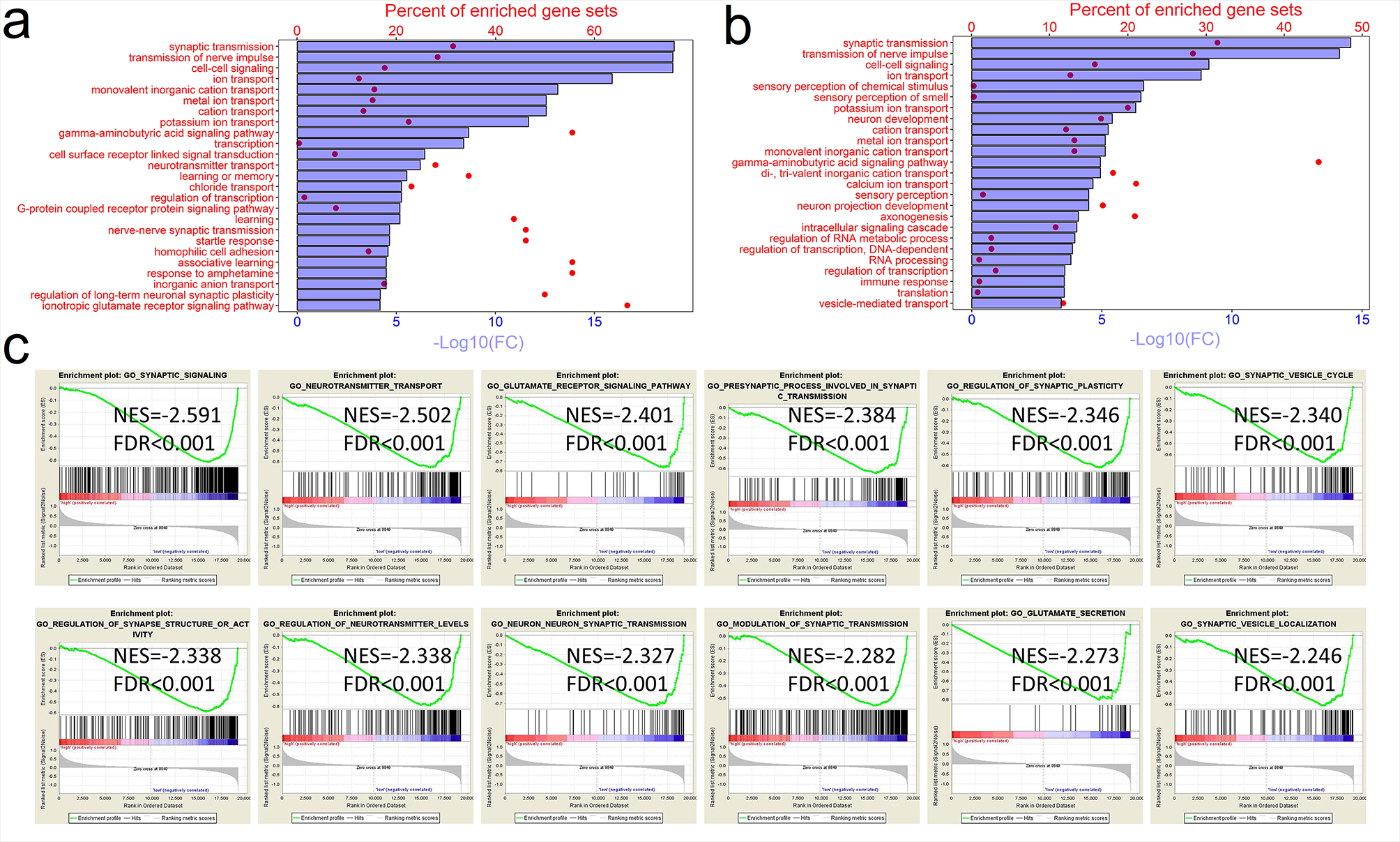
**

**Figure S3.** Biological function of AP score. **(a)** Top 25 GO terms enriched by down-regulated DEGs [Log10(FC) < -1.5] of AP score. **(b)** Top 25 GO terms enriched by negatively correlated (r < -0.6) genes of AP score. **(c)** GSEA results based on decreasing AP score.

**Table S1** Clinical characteristics of the AP score in the training cohort and validation cohort.

| Patient characteristics | Training cohort (TCGA RNA-seq) | | | Validation cohort (GSE16011) | | |
| --- | --- | --- | --- | --- | --- | --- |
|  | AP score  Low  (n=233) | AP score  High  (n=233) | P value | AP score  Low  (n=92) | AP score  High  (n=92) | P value |
| Age (year) |  |  | **<0.0001** |  |  | **0.0011** |
| <60 | 217 | 132 |  | 72 | 50 |  |
| ≥60 | 16 | 101 |  | 20 | 42 |  |
| Gender |  |  | 0.5113 |  |  | 0.7521 |
| Male | 131 | 139 |  | 31 | 28 |  |
| Female | 102 | 94 |  | 61 | 64 |  |
| Grade |  |  | **<0.0001** |  |  | **0.0002** |
| II | 113 | 12 |  | 12 | 1 |  |
| III | 89 | 70 |  | 12 | 4 |  |
| IV | 4 | 139 |  | 68 | 87 |  |
| Histology |  |  | **<0.0001** |  |  | **0.0002** |
| Astrocytoma | 98 | 65 |  | 24 | 5 |  |
| Glioblastoma | 4 | 139 |  | 68 | 87 |  |
| Oligoastrocytoma | 70 | 14 |  | - | - |  |
| Oligodendroglioma | 34 | 3 |  | - | - |  |
| IDH1 |  |  | **<0.0001** |  |  | **0.0014** |
| Mutant | 211 | 45 |  | 31 | 11 |  |
| Wild type | 22 | 188 |  | 46 | 61 |  |
| MGMT |  |  | **<0.0001** |  |  | **-** |
| Methylated | 192 | 103 |  | **-** | **-** |  |
| Unmethylated | 40 | 101 |  | **-** | **-** |  |
| Transcriptional subtype |  |  | **<0.0001** |  |  | **<0.0001** |
| Neural | 57 | 6 |  | 8 | 10 |  |
| Proneural | 103 | 29 |  | 25 | 4 |  |
| Classical | 2 | 75 |  | 22 | 48 |  |
| Mesenchymal | 2 | 84 |  | 4 | 25 |  |

**Continued on next page**

| Patient characteristics | Validation cohort (REMBRANDT) | | | Validation cohort (TCGA GBM) | | |
| --- | --- | --- | --- | --- | --- | --- |
|  | AP score  Low  (n=124) | AP score  High  (n=124) | P value | AP score  Low  (n=188) | AP score  High  (n=188) | P value |
| Age (year) |  |  | - |  |  | 0.9177 |
| <60 | - | - |  | 90 | 88 |  |
| ≥60 | - | - |  | 98 | 100 |  |
| Gender |  |  | - |  |  | 0.0892 |
| Male | - | - |  | 108 | 125 |  |
| Female | - | - |  | 80 | 63 |  |
| Grade |  |  | **<0.0001** |  |  | **-** |
| II | 34 | 12 |  | - | - |  |
| III | 24 | 15 |  | - | - |  |
| IV | 33 | 75 |  | 188 | 188 |  |
| Histology |  |  | **<0.0001** |  |  | - |
| Astrocytoma | 67 | 29 |  | - | - |  |
| Glioblastoma | 57 | 95 |  | 188 | 188 |  |
| IDH1 |  |  | **-** |  |  | - |
| Mutant | - | - |  | 0 | 0 |  |
| Wild type | - | - |  | 188 | 188 |  |
| MGMT |  |  | **-** |  |  | 0.5709 |
| Methylated | - | - |  | 58 | 54 |  |
| Unmethylated | - | - |  | 78 | 86 |  |
| Transcriptional subtype |  |  | **<0.0001** |  |  | **<0.0001** |
| Neural | 26 | 4 |  | 34 | 12 |  |
| Proneural | 18 | 13 |  | 51 | 8 |  |
| Classical | 8 | 59 |  | 60 | 56 |  |
| Mesenchymal | 5 | 19 |  | 30 | 106 |  |

P value was calculated by Chi-squared or Fisher’s exact test depending on patient counts in each group. Missing value was not included in the table.

**Table S2** Correlation and prognostic value of each enriched gene set in the training cohort.

| Gene sets | Log-rank test | Correlation with AP score (Pearson correlation) | |
| --- | --- | --- | --- |
|  | P | r value | P |
| HALLMARK_INTERFERON_GAMMA_RESPONSE | **<0.001** | 0.726 | **<0.001** |
| HALLMARK_G2M_CHECKPOINT | **<0.001** | 0.640 | **<0.001** |
| HALLMARK_EPITHELIAL_MESENCHYMAL_TRANSITION | **<0.001** | 0.800 | **<0.001** |
| HALLMARK_INTERERON_ALPHA_RESPONSE | **<0.001** | 0.655 | **<0.001** |
| HALLMARK_INFLAMMATORY_RESPONSE | **<0.001** | 0.645 | **<0.001** |
| HALLMARK_APOPTOSIS | **<0.001** | 0.819 | **<0.001** |
| GO_MITOTIC_SISTER_CHROMATID_SEGREGATION | **<0.001** | 0.615 | **<0.001** |
| GO_NUCLEAR_DIVISION | **<0.001** | 0.624 | **<0.001** |
| GO_ADAPTIVE_IMMUNE_RESPONSE | **<0.001** | 0.640 | **<0.001** |
| GO_IMMUNE_RESPONSE | **<0.001** | 0.655 | **<0.001** |
| GO_CELL_CYCLE_G1_S_PHASE_TRANSITION | **<0.001** | 0.713 | **<0.001** |
| GO_INFLAMMATORY_RESPONSE | **<0.001** | 0.644 | **<0.001** |
